# Supplementary material for: A global exploratory comparison of country self-citations 1996-2019
Source: PLoS One. 2023 Dec 29;18(12):e0294669. doi: 10.1371/journal.pone.0294669 (PMC10756561; doi:10.1371/journal.pone.0294669)
Supplement: S1 File — (PDF) [file pone.0294669.s001.pdf]

A global exploratory comparison of country self-citations 1996-2019

Alberto Baccini Eugenio Petrovich

Supporting Information S1 – Supplementary Figures

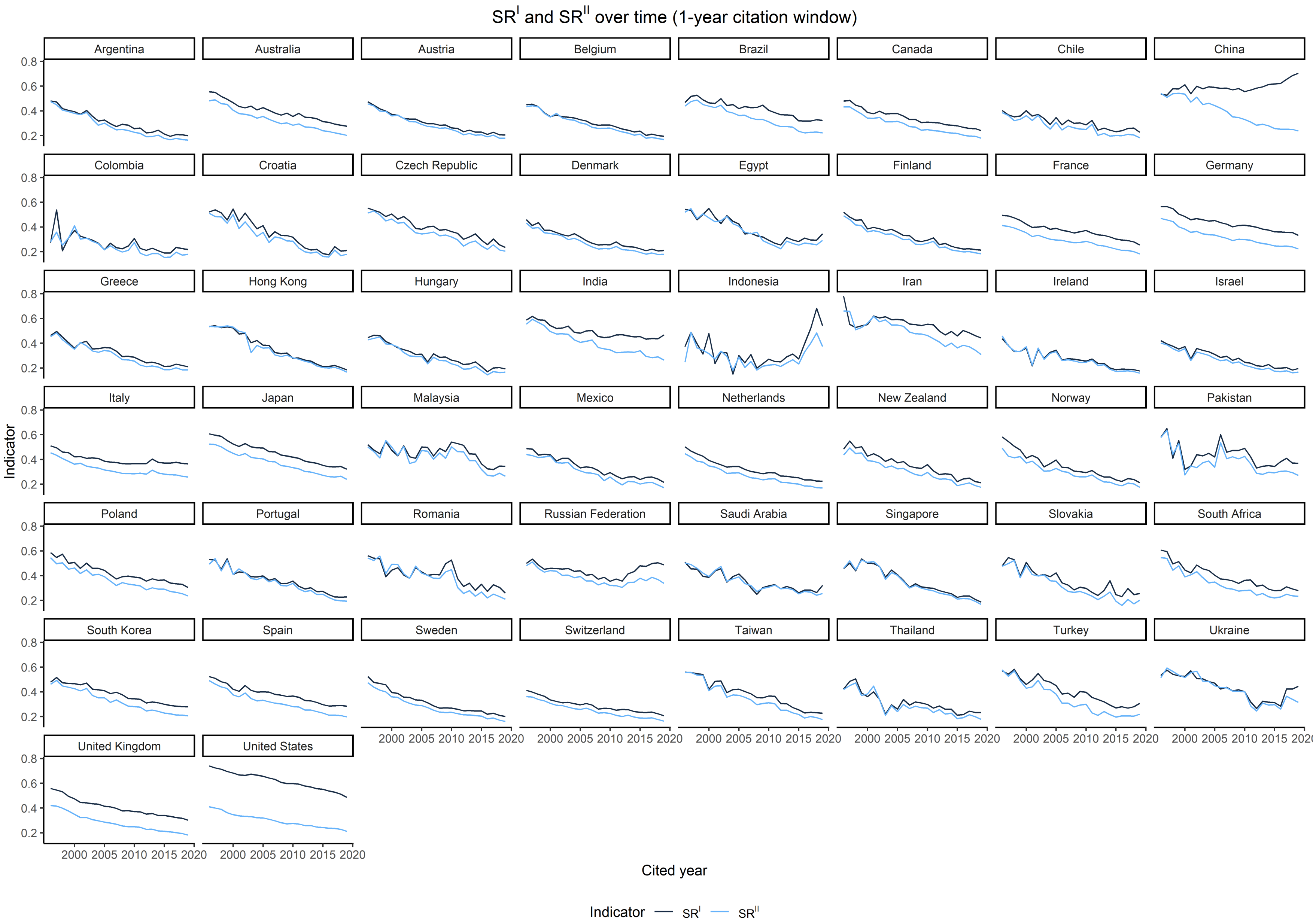

Figure 1: SR^I and SR^{II} over time (1-year citation window)

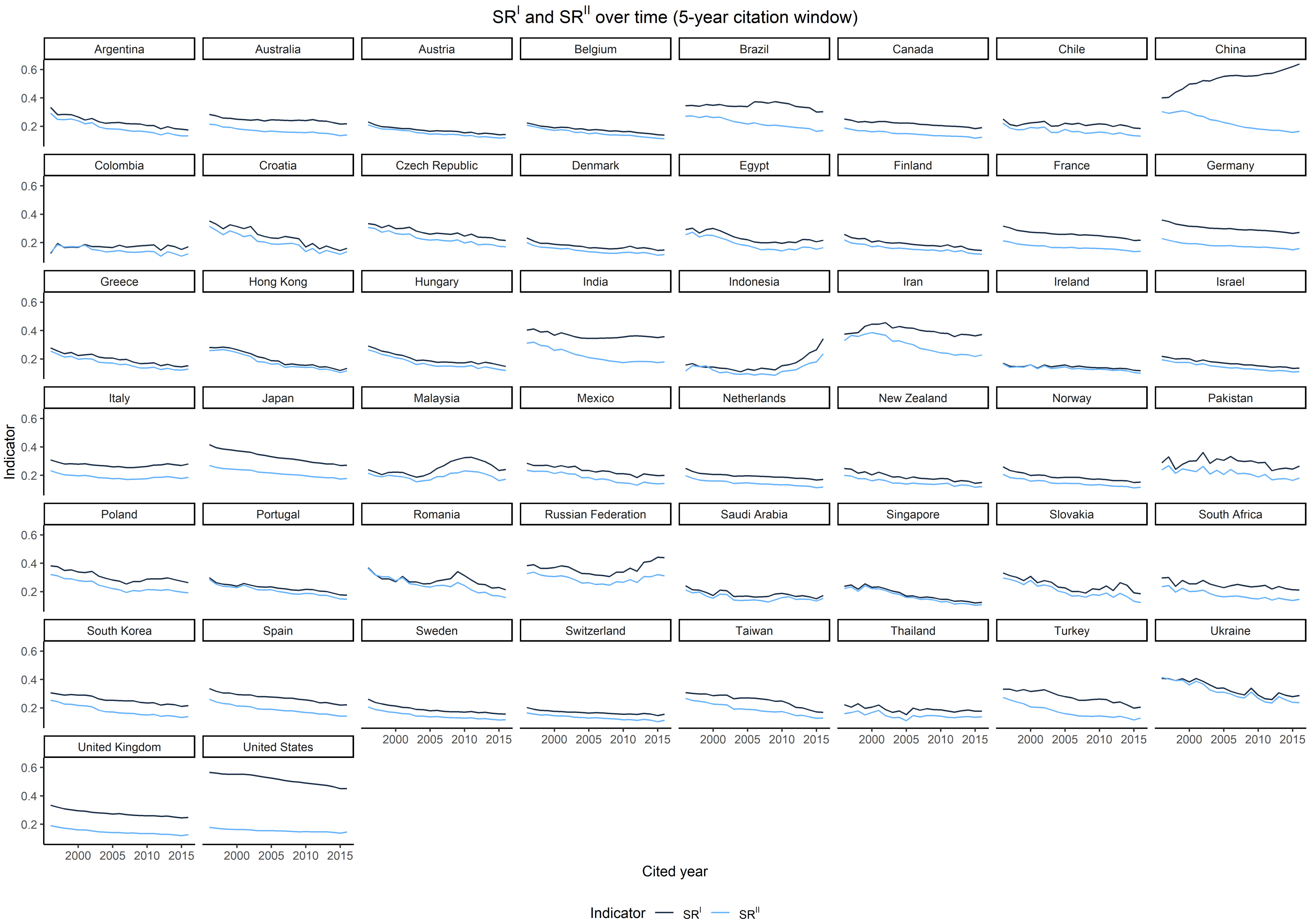

Figure 2: SR^I and SR^{II} over time (5-year citation window)

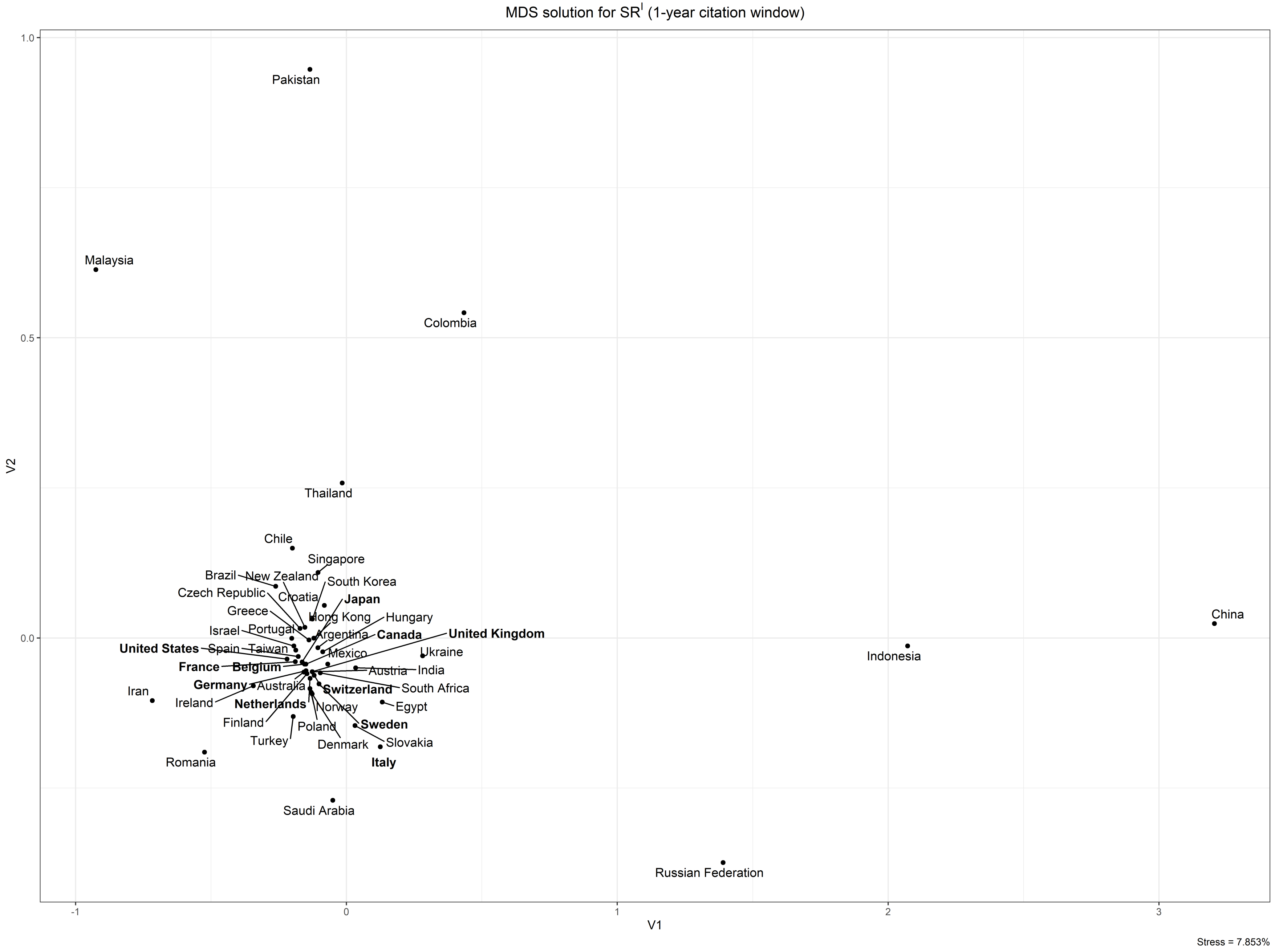

Figure 3: MDS solution for SR^I (1-year citation window)

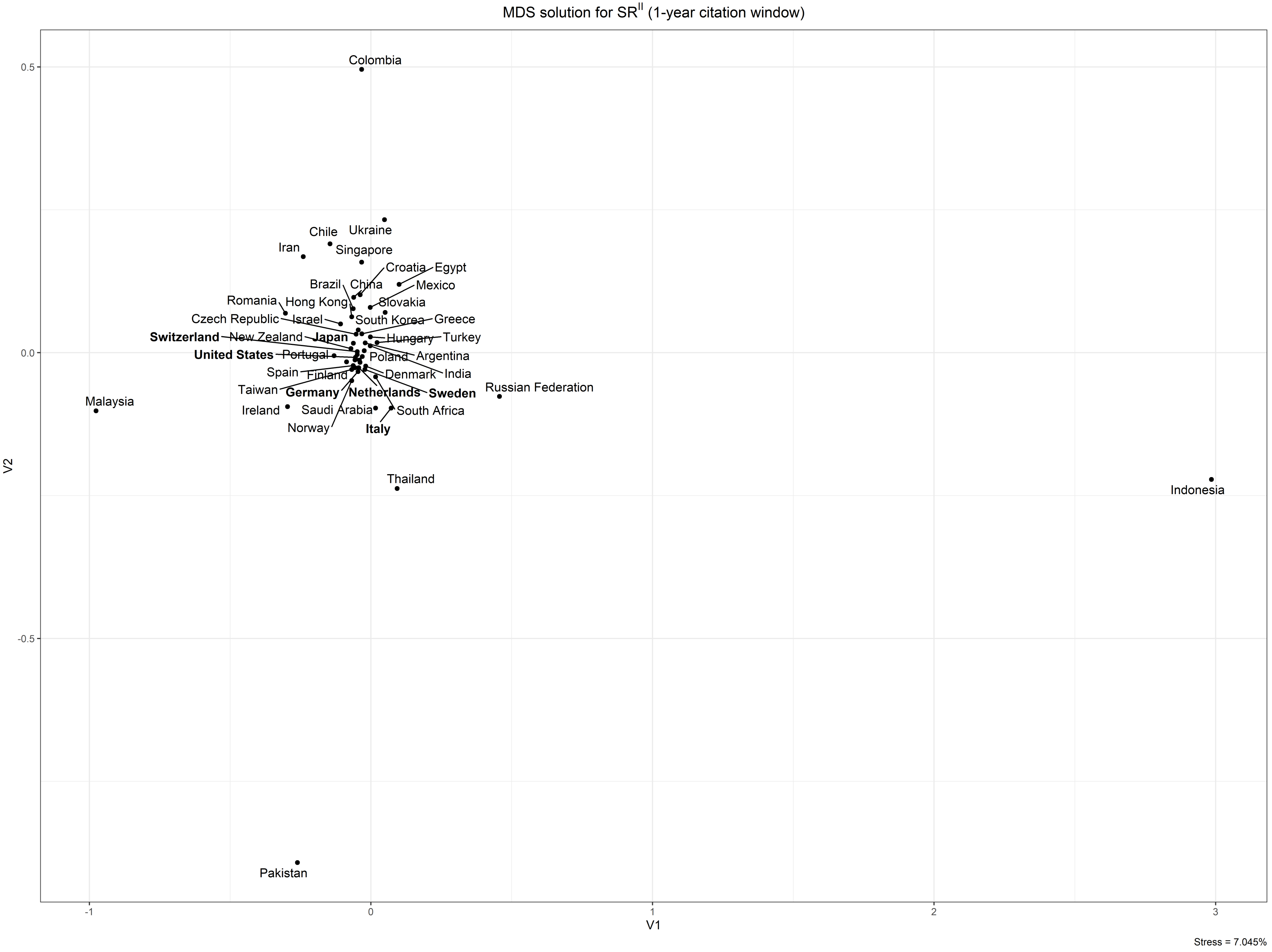

Figure 4: MDS solution for SR^{II} (1-year citation window)

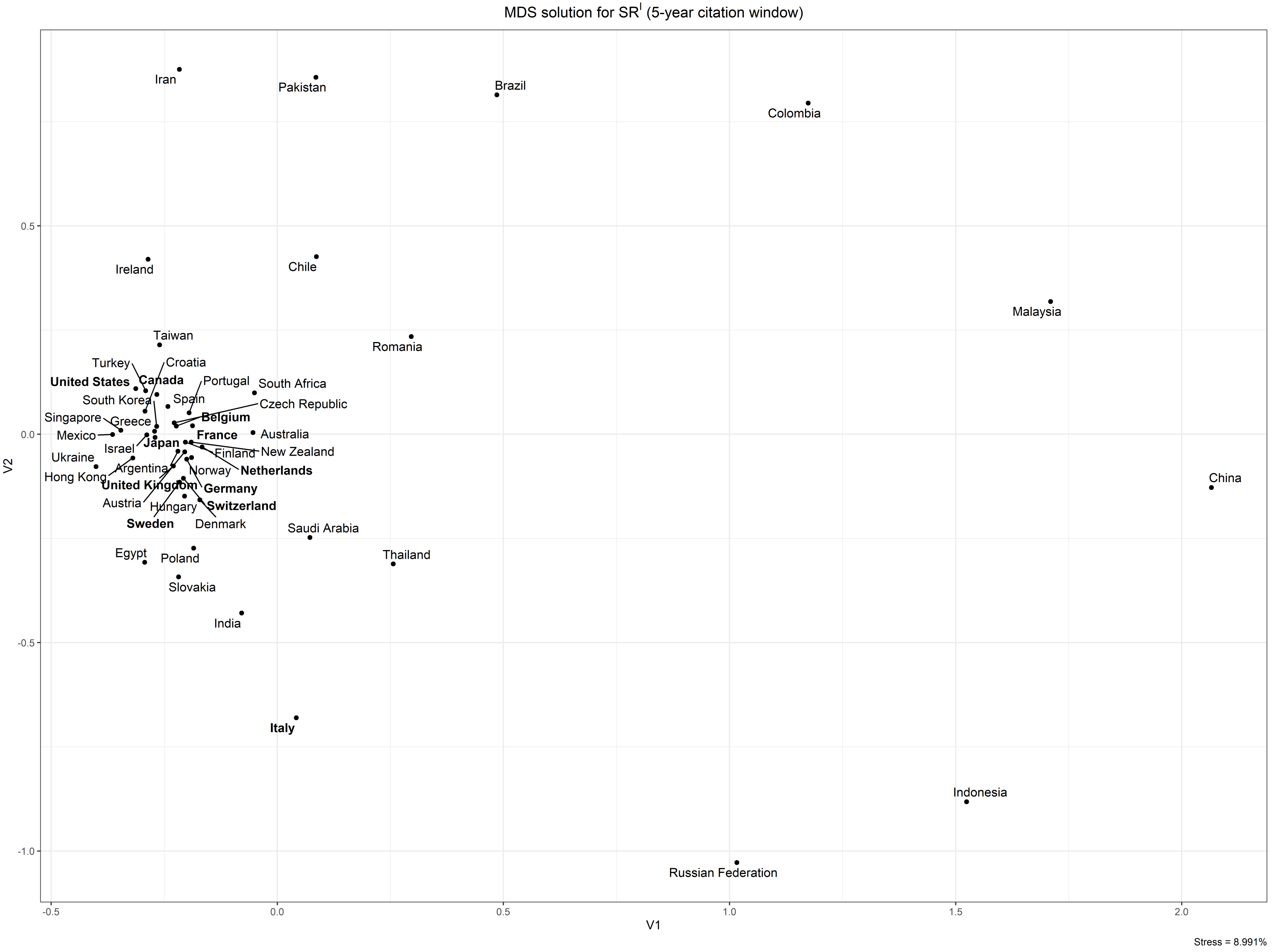

Figure 5: MDS solution for SR^I (5-year citation window)

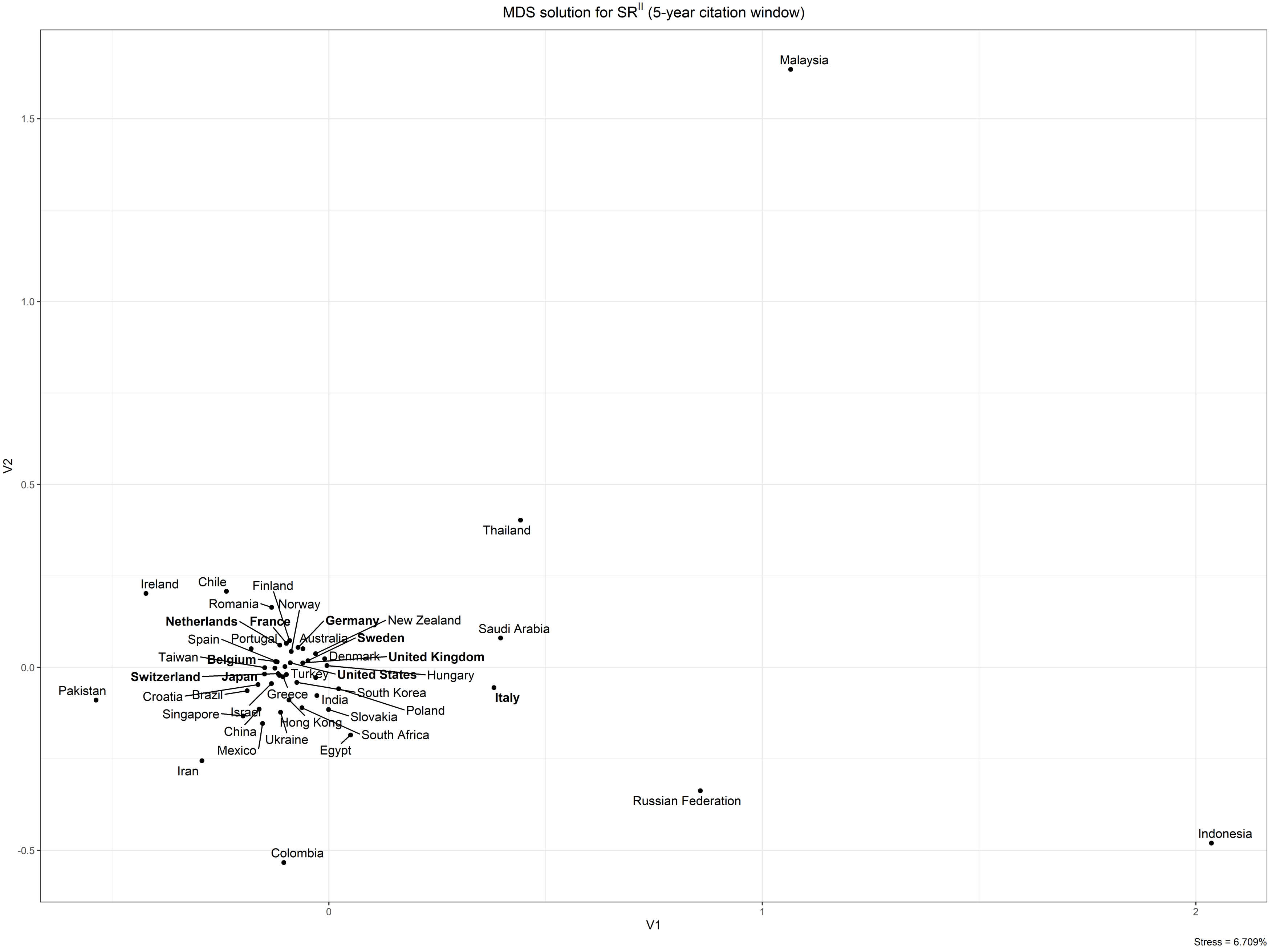

Figure 6: MDS solution for SR^{II} (5-year citation window)

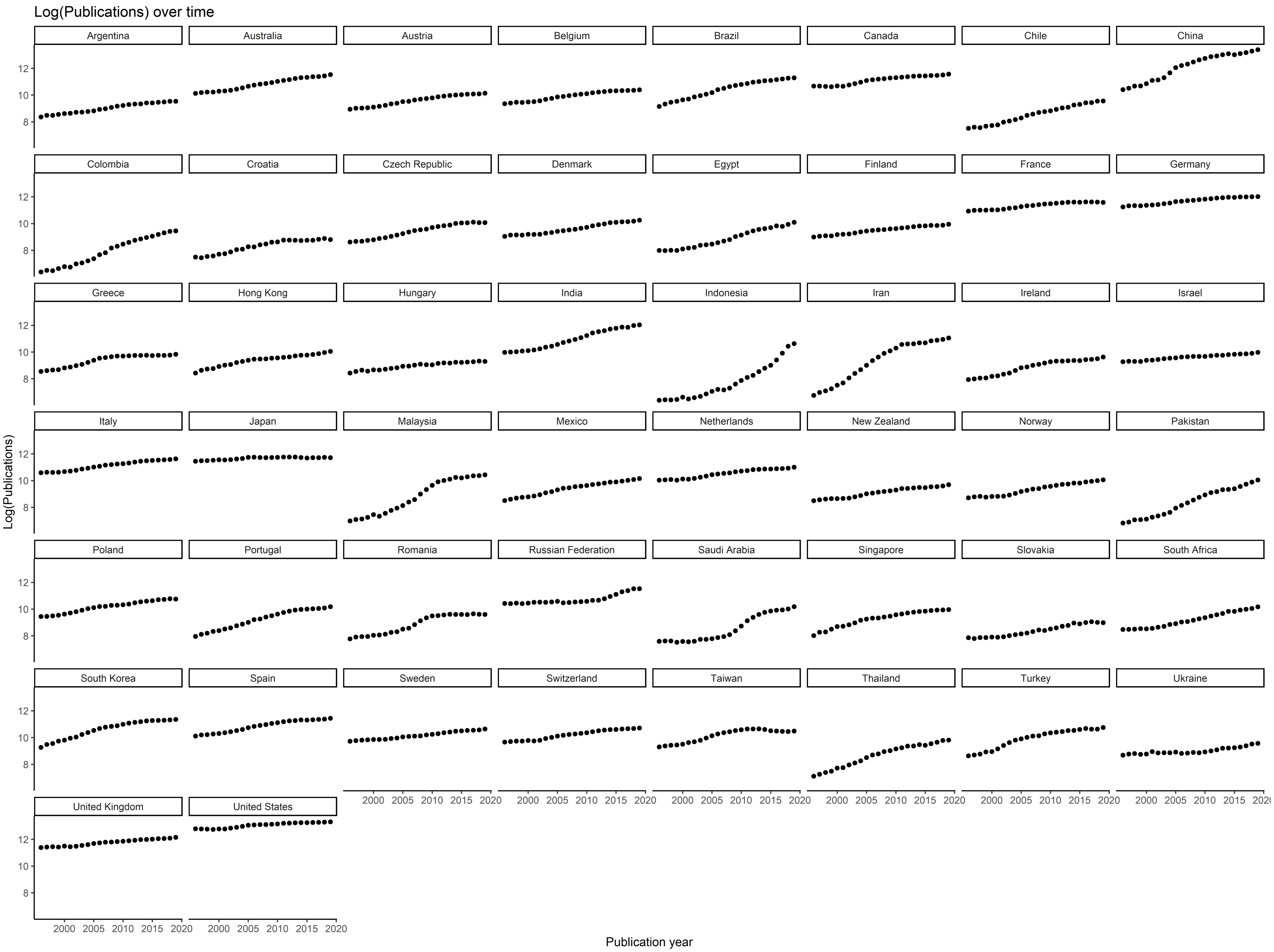

Figure 7: Publication output over time (log scale)
