# Supplementary material for: A global exploratory comparison of country self-citations 1996-2019
Source: PLoS One. 2023 Dec 29;18(12):e0294669. doi: 10.1371/journal.pone.0294669 (PMC10756561; doi:10.1371/journal.pone.0294669)
Supplement: Attachment — Submitted filename: Responses to the Reviewers.docx [file pone.0294669.s003.docx]

**Responses to the Reviewers**

*Please find our answers to the Reviewers’ remarks in the following. Reviewers’ comments are in standard formatting, our answers are in light blue. Changes to the paper’s text are indented, with added lines highlighted in yellow.*

*Please note that we modified the affiliation of the second Author.*

# ***Reviewer #1***

The issue is relevant. Indeed, the centrality of metrics in the research system, from individual researchers' careers to institutional rankings and country assessments, has heightened these concerns. The study examines changes in self-citation rates in 50 countries from 1996 to 2019 and finds that, overall, self-citation trends appear to be declining. However, some countries show anomalous behaviour, with self-citation trends that differ significantly from the "standard" countries. The article is well presented and respects the structure of a scientific article. The analysis focuses on a phenomenon of self-citation with a discussion of bibliometrics at the meso level. The discussion focuses on science policy, in line with the problem.

It is argued that policies focused on citation performance significantly affect the behaviour of scientists and calls for caution in the use of bibliometric indicators, as they can become active components of the research system and change the behaviour of scientific communities at the country level.

The state of the art allows the definition of self-citation and self-reference. It also presents the formal framework within which the study is carried out.

The method is based on the analysis of time series with a differentiation between countries in the context of self-citation, which allows a more detailed analysis of this issue. The literature is well used in the construction of the method. Figure 1, although brief, contributes to a good understanding of the article. The section on design indicators is explicit about implementation.

A sensitive part of the study concerns the comparison of time series. The chosen approach allows the calculation of an adaptive dissimilarity index between two time series. This makes it possible to capture both the raw dissimilarity and the dissimilarity in the temporal correlation behaviour.

We thank the Reviewer for appreciating the article and their supportive comments. We are happy that they agree on the relevancy of the issue investigated in the paper.

The figures could be improved. Figures 3 and 4 and the legend need to be enlarged to make them easier to read. The text of Figure 5 should be enlarged.

We increased the font size of both the labels and the caption in Figures 3, 4, 5 (now 6) and 6 (now 7) to make them more readable. Note that we added a new Figure (the new Figure 5) to report new results about the correlation between self-citation rates and publication output.

In the discussion, the presentation of the different policies per country is relevant. The conclusion is well balanced with the limitations of the study.

We thank the Reviewer for the positive assessment of discussion and conclusion.

The results obtained and the discussion invite a more detailed conclusion on the limitations and perspectives of this study, in order to offer a deeper reflection on self-citation in a global context and on its nature.

In the Results section, we added a new Figure showing the relationship between self-citation rate and publication trends, in order to control for the effect of publication behavior on self-citation behavior. The following lines were added:

[after line 431] Both indicators result to be highly correlated with the logarithm of the publication output for most of the countries (Fig. 5). Notably, the relationship between the self-citation rate (of both Types) and scientific production is of inverse proportionality: across most of the countries observed, an increase in scientific output is associated with a decrease in the self-citation rate. This contrasts with the direct proportionality found in prior studies [30, 40, 41, 46]. Interestingly, however, a few countries deviate from this general pattern, exhibiting either positive correlation (China for $SR^{I}$, Indonesia on both $SR^{I}$ and $SR^{II}$) or mild correlation coefficients (Colombia, Egypt, India, Iran, Italy, Malaysia, Pakistan, Romania, Russian Federation, Saudi Arabia, Thailand, Ukraine). The self-citation rate trend within this latter group of anomalous countries cannot be effectively predicted based solely on the trend of scientific production. (The scientific output trends of the 50 countries are reported in the Supporting Information.)

[after line 450] Note that the countries in the two latter zones are identical to those showing anomalous correlations with trends in scientific output.

[after line 509] Note that the anomalous self-citation rates of these countries cannot be explained by the trend of their scientific production, as evidenced by the low correlation coefficients found (Fig. 5). This suggests to search for specific perturbations of the citation behavior.

In the Supporting Information, an extra figure (S7) was added with the publication trends of the 50 countries.

To propose some lines of future research, we added the following lines to the Conclusions:

In future research, it will be interesting to further investigate into the effects of policy pressure. For instance, by examining whether research fields characterized by varying epistemological, social, and institutional features respond differently to science policies. Or by investigating whether science policies may affect countries’ mix of scientific output, inducing specialization in areas rewarded by citation metrics (e.g., the medical sciences) at the expense of less “performing” areas (e.g., the humanities).

The bibliography is well constructed and used. DOIs are present.

Some typographical and spelling errors need to be corrected and require a second reading: l 129, l 436, l 574, l 132, ...

We thank the Reviewer for spotting the typos. We read throughout the text and corrected them.

# ***Reviewer #2***

The main purpose of this work is described in line 43 of the Introduction: “In this study, the trends of country self-citations in 50 countries over the world are investigated in order to reveal groups of countries characterized by similar self-citation behavior in time”.

Self-citations are a key topic in evaluative bibliometrics. Country self-citations are a very useful indicator at the macro level to highlight certain phenomena that can be traced to structural elements of a country's research infrastructure or to national policies adopted within it.

The topic addressed is, therefore, extremely relevant and highly topical. However, although well written, easy and clear to understand, the paper has some remarkable methodological weaknesses which, unfortunately, undermine its results and therefore fail to substantiate the findings.

We thank the Reviewer for agreeing with us on the relevancy of the topic and acknowledging the clearness of the study. We appreciate the Reviewer for posing insightful objections to our approach, which have prompted us to engage in a more profound reflection on our methodology and, also, produce insightful new results. In the following sections, we address the Reviewer's methodological concerns and respond to their criticisms.

My major concern about the methodological framework adopted by the authors is represented by the choice to use self-citation and not self-referencing.

Already in the abstract, the authors state that: “12 countries (…) exhibit anomalous trends of self-citations. We argue that these anomalies should be attributed to the aggressive science policies adopted by these countries in recent years, which are all characterized by direct or indirect incentives for citations. Our analysis confirms that when bibliometric indicators are integrated into systems of incentives, they are capable of affecting rapidly and visibly the citation behavior of entire countries”.

In line 513 of the Discussion: “All these policies are characterized by direct or indirect incentives that may create room for the strategic use of self-citations. SRI and SRII (the two indicators on which the whole analysis is based), therefore, seem to be sensitive to policy-induced perturbations of the citation habits”.

The point is that “Habits” refers to “active” and not “passive” behaviours, therefore, their analysis cannot be based on self-citations but on self-references. Note that the word “behaviour” appears 19 times in the paper (5 in the Conclusions).

I think it is a very serious fault also because the authors do not deny this evidence, and at the very beginning (line 7 of the introduction) they say, “As for author self-citations, also country self-citations should be distinguished from country self-references”. “Notably, country self-references depend only on the behavior of the researchers from the country. By contrast, country self-citations are determined also by the behavior of other citing countries”.

How can the authors investigate the possible occurrence of "opportunistic behavior" through the use of an indicator tracking the behavior of entities not subject to the "treatment" (the incentive)?

More importantly, how can such a choice be justified considering that instead, there is an indicator that tracks the behavior of all and only those entities subject to the "treatment"?

We thank the Reviewer for their attentive reading and for advancing an objection that allows us to better explain our choice of focusing on self-citation rates rather than self-reference rates. The Reviewer’s reasoning seems *prima facie* compelling: if the researchers that act opportunistically modify their referencing behavior, the focus of the study should be on the citations they *give* (i.e., the references), rather than on the citations they *receive*. A closer look, however, shows that such a reasoning, albeit intuitive, is based on a misunderstanding of the notion of self-citation. Moreover, we can show that the alternative indicator suggested by the Reviewer – the self-reference rate – is less effective than our indicator in tracking the strategic use of citations in response to incentives.

The key point is that “self-reference” and “self-citation” are two different names for the *same* citation link in the citation network. Let author $A$ produce two publications $p_{1}$ at time $t_{2}$ and $p_{2}$ at time $t_{4}$, and be $p_{c}$ the publication cited by $p_{1}$and $p_{a}$, $p_{1}$, and $p_{b}$ the publications cited by $p_{2}$, as in the following diagram (publications from the same author $A$ are in shaded grey):

Let us focus on the citation link ${(p}_{2}\to p_{1})$. The link was materially generated at $t_{4}$ in publication $p_{2}$ as the product of a referencing act of $A$, i.e., as an effect of her behavior (an active choice taken during the writing of the paper). Now, it is clear from the diagram that calling the link ${(p}_{2}\to p_{1})$ a self-reference or a self-citation is only a matter of perspective: if we focus on $p_{2}$, the link counts as a *self-reference* of author $A$, whereas if we focus on $p_{1}$, it counts as a *self-citation* of author $A$. This small example shows that, in the case of self-citations, the distinction between a citation *given* and a citation *received* by an author (or any other bibliographic entity considered) is only a matter of viewpoint. Therefore, the *counts* of self-references and self-citations coincide, provided that an adequate citation window is chosen. This means, in turn, that an author (or any other bibliographic entity considered) can *intervene* on its citation counts by modifying their referencing behavior: their self-references will turn into self-citations when seen from the viewpoint of the cited publications.

Still, one may object that the interesting indicator are *not* self-citations/self-references *per se* but rather self-citations/self-references divided by some relevant quantity, i.e., self-citation *rates* (the indicator chosen in our study) and self-reference *rates* (the indicator that the Reviewer seems to have in mind), respectively. (Note that self-reference rate $SBR$ is discussed at length in lines 100-121 of the paper). Here it is true that a difference emerges: the self-citation rate and self-reference rate of the same bibliographic entity do *not* necessarily coincide. The difference, however, does not depend on the *numerator* of the ratio (as self-citations and self-references are the same links in the citation network as we have just seen) but on the *denominator*. In the self-citation rate, in fact, the denominator is the total number of *citations* received by the bibliographic entity; in the self-reference rate, by contrast, the denominator is the total number of *references* (i.e., citations given) by the bibliographic entity (as explained in detail in lines 100-121 of the paper). The difference is clear from the diagram above: author $A$ has a self-reference rate of $\frac{1}{4}=25\%$ but a self-citation rate of $\frac{1}{2}=50\%$.

Importantly, this example helps us showing why self-citation rates are in fact a *better* indicator of strategic behavior than self-reference rates. If we assume that $p_{1}$ had 0 citations at time $t_{3}$, author $A$ can efficiently increase the citation count of $p_{1}$ by adding to the bibliography of $p_{2}$ just 1 reference (to $p_{1}$) at $t_{4}$. This action has a relatively small cost in terms of self-reference rate increase (from $0\%$ to $25\%$) but a tangible effect in terms of the author total citation count, which, at time $t_{5}$ will have 2 citations instead of only 1 ($+100\%$).

Now, self-reference rates (at least at the level of the individual paper) can be easily controlled by reviewers or editors, who can spot an anomalous amount of self-references in the bibliography of a paper. Indeed, certain journals explicitly warn the referees to check for excessive self-referencing in the submissions. But in order to artificially increase citation counts, an author does not need to *significantly* alter her self-reference rate: it is sufficient to add one or two self-references pointing to smartly chosen papers and bury them in enough long reference lists. These self-references will boost the citation counts of the cited papers and thus the citation metrics of the author.

At the same time, however, these few self-references will inescapably increase also the total number of self-citations of the author. And, in order to renormalize her self-citation rate, our author should be able to control the denominator of the formula, i.e., all the citations she receives. Unfortunately for her, however, she cannot control the external citations provided by other authors (save for establishing a citation exchange system). This means that if total citations do not compensate for the excessive self-references/self-citations, the self-citation rate of the author will inevitably increase, allowing us to detect an anomaly. Anomalous self-reference rates, by contrast, can easily disappear in long reference lists.

To better clarify the advantages of the self-citation rate over the self-reference rate, we added the following lines after line 121:

Note that, in general, authors have more control over their self-reference rate than their self-citation rate, as they can manage more easily the citations they give (i.e., the denominator of the self-reference rate), than the citations that they receive (i.e., the denominator of the self-citation rate). In fact, authors interested in artificially inflating their citation metrics can effectively conceal strategic self-references within long reference lists and keep their self-reference rate low. Still, if they are not able to attract enough external citations to offset the impact of their strategic self-references, their self-citation rate will inevitably rise. This shows that the self-citation rate is more effective in revealing strategic self-citation behavior than the self-reference rate.

Even more difficult to understand the choice to adopt self-citations instead of self-references, considering the computational complications involved. Line 69: “The computation of self-citations requires the definition of: (i) a publication window …; b) a citation window”. Line 103: “The computation of self-reference requires only a publication window for the set of citing sources”. And also, in line 321: “A citation window is required to correct for the fact that older publications have more time to accumulate citations”. Not required if self-references are used in place of self-citations.

We regard rigorousness in defining bibliometric indicators as a strength rather than as a flaw in our approach. Moreover, the simplicity of definition of self-reference rate is only apparent. Indeed, also a yearly computed self-reference rate implicitly assumes a publication window of one year and a citation window that goes back until the year of the oldest reference appearing in the bibliographies of papers published in the publication window. To be fully rigorous, hence, both a publication window and a reference-window should be specified also in the case of self-reference rate, as we did with the self-citation rate. To clarify this, we added the following lines after Equation 4:

Note that a rigorous definition of this indicator would require the specification of a reference window, analogous to the citation window used in the self-citation rate. The studies based on the self-reference rate (e.g., [16]) usually assume a reference window that is extended to the oldest publication year of the references considered, but, in principle, other reference windows are possible.

We highlight that citation windows are common in bibliometrics: for instance, they are used in the definition of the Journal Impact Factor.

Note that, as we were aware that the length of the citation window was a further parameter, in the Supplementary Information we replicated all the analyses using different citation windows, obtaining qualitatively similar results (as reported in lines 350-355 of the manuscript).

In summary, I believe that every sentence in the paper should be reconsidered in light of this "original sin."

In order to avoid misunderstanding and better clarify the relationships between self-citations, self-references, and their respective rates, the following paragraph were modified:

Thes lines 36-42 were removed: ~~As for author self-citations, also country self-citations should be distinguished from country self-references. The former shows how much of the citation impact of a country is generated from within the country itself, whereas the latter shows how much the publications of a country refer to scientific knowledge produced within that country. Notably, country self-references depend only on the behavior of the researchers from the country. By contrast, country self-citations are determined also by the behavior of other citing countries [16].~~

[lines 109-110]: ~~Analogously,~~ $R_{E,y}$~~indicates the total number of citations received by cited items from citing sources produced by~~ $E$ ~~in the year~~$y$~~.~~ Analogously, $R_{E,y}$indicates the total number of citations given (i.e., references) by $E$ in the year$y$ to documents published before.

For instance, “The country self-citation rate results to be positively correlated with the publication output of a country”. I think the correct statement is, "The country self-reference citation rate results to be ...". The more output a country produces, the more chance the country has to self-cite.

The result we refer to appeared in the cited publication [30], where the indicator is the self-citation rate and not the self-reference rate, as it is clear from the following quote of the cited publication: “The rate of country self-citations was calculated by dividing the total number of country self-citations, according to the country of residence of the corresponding author given by SCImago, by the total number of citations for each country” ([30, p. 695])

Or: “The odds-ratio ON,y measures to what extent the country relative preference to cite its own publications”. Wrong: this refers to self-referencing.

As before, the interpretation of the odds-ratio that we report comes from the cited publication [41], where it is written that: “This probability ratio indicates the odds favouring an author’s choosing to cite a publication from his or her own country, relative to what the odds would be if he had chosen at random from the world’s literature.” ([41], p. 294).

Even more in the Conclusions, two examples for all:

• “Self-citation, in particular, has persistently been indicated as among the easiest strategies available to scientists for artificially boosting their citation-related performance indicators, raising the question of whether scientists, under pressure, do indeed attempt to game citation scores by increasing self-citation”.

• “Scientists do indeed respond to the new climate of incentives by altering, among other things, their citation habits. Policy pressure seems therefore capable of affecting rapidly and visibly the citation behavior of entire countries”.

As discussed at length above, a self-citation is a self-reference seen from the viewpoint of the cited publication and, alternatively, a self-reference is a self-citation seen from the viewpoint of the citing publication. Hence, there is no contradiction in saying that researchers can use self-citations strategically. We add that very similar words are used in a recent review paper on self-citations (reference [6]): “The issue is whether, under pressure, scientists and scholars do indeed increase self-citation artificially to boost their potential for rewards (including appointments, promotions, and funding).” ([6], p. 1121) The bibliometrics community seems to have reached a consensus on considering self-citations in these terms.

Other points:

1) “A citation is considered a country self-citation when the intersection between the set of the countries of affiliation of the author(s) of the cited publication and the set of the countries of affiliation of the author(s) of the citing publication, is not empty”. This may be acceptable only if the self-citation is attributed solely and exclusively to the “intersection” and not to all authors' countries. As a consequence, one of the two indicators used by the authors (country self-citations of Type I) is wrong, being affected by systematic error.

As explained at length in the methodological sections of the paper, there is no unique method for computing country self-citations for international publications, as there is no unique method for computing author self-citations for multi-authored publications. Our choice of attributing country self-citations of Type I to all *the* country participating in the cited/citing publications is in line with Elsevier SciVal’s methodology (see Elsevier. Research Metrics Guidebook; 2018. Available from: <https://www.elsevier.com/__data/assets/pdf_file/0020/53327/ELSV-13013-Elsevier-Research-Metrics-Book-r5-Web.pdf>.). Preferring such a method increases the replicability of our study because other researchers can have access to Elsevier’s data easily. Another option discussed in the bibliometric literature (see e.g. reference [43] in the paper and our comments on the lines 156-159) is to adopt a *fractional* counting, when the self-citation is equally split among the collaborating countries. This option, however, has the drawback of producing non-integer citation counts that are more difficult to interpret. Still, we note that there is no proposal in the bibliometric literature of attributing citations to “intersections of countries” as proposed by the Reviewer. The reason is that such a method would produce a huge plethora of artificial bibliographic entities: : in our case 1,225 intersections representing each a pair of countries, 19,600 intersections representing each a triple of countries; 230,030 intersections representing each a quadruple of countries, and so on. We consider therefore the Reviewer’s suggestion not only as not in line with bibliometric data providers’ established practices, but also as non-viable in principle.

Regarding the supposed “systematic error” in country self-citations of Type I mentioned by the Reviewer, we note that country self-citations of Type II were introduced in the study also for better controlling the effect of international publications (see the example and discussion in lines 244-276 of the paper, plus Figure 2 and Table 1). Still, the indicator based on Type I country self-citations, $SR^{I}$ or extensive self-citation ratio, remains interesting in our view because, compared to $SR^{II}$, it tracks also the internal exchange of citations of a country. Finding that some countries, such as China, are characterized by different trends in the two indicators is thus a relevant result of the paper. Removing $SR^{I}$ would unnecessarily limit the findings of the study. Clearly, we remain open to the possibility that future research might offer an alternative explanation for these trends.

Among others, the authors use this indicator in order to capture “the effect of citation clubs at the country level” … really suggestive, but honestly, at least a bit far-fetched to be achieved with such an indicator.

Please note that we do not claim that $SR^{I}$ captures *only* the effect of citation clubs. We claim that it is sensitive *also* to citation clubs (see lines 286-290). To increase clarity, we modified the following sentence on lines 314-317:

Even if country self-citations of Type I are affected by citations coming from other countries, as seen above, they remain interesting because ~~and~~ they allow capturing, among other things, the effect of citation clubs at the country level, i.e., the strategic exchanges of citations between researchers in the same country that are not co-authors.

2) “In static analyses of country self-citations, indicators encapsulating both publications and country self-citations are useful, but they become problematic when the temporal dimension is considered”. [Therefore] … the most suitable indicator is the self-citation ratio only”. This is true, but if you want to capture truly anomalous events (possible strategic behaviours), you must necessarily analyse self-citations together with output. As stated by the authors, if there is a clear causal nexus between output and self-citations, then an "entity" E with an anomalous pattern in terms of output will be able to register, ceteris paribus, an anomalous pattern in terms of self-citations as well. This applies to individual scholars and also to countries.

We respond to points 2 and 3 together below.

3) Again, about the causal link between output and self-citations, it is clear that the authors' interpretation of anomalous patterns on self-citations, I guess, applies to output as well. Are we sure that this is “citation gaming”? Doesn't it all depend on the expansion of the output of these countries? The methodological approach proposed by the authors is inadequate to answer that.

We extend our gratitude to the Reviewer for recommending a more in-depth exploration of the connection between self-citation rates and output. This analysis has enabled us to generate supplementary findings that are not only inherently valuable but also serve to reinforce our conclusions.

In particular, we analyzed the correlation between the trend in the publication output, on the one hand, and the two self-citation rates, on the other hand. The results (see Figure below) clearly indicate that the standard relationship between the two variables, i.e., the relationship found for most countries, is *not* of direct proportionality as found in prior studies, but of inverse proportionality. Even more interestingly, 12 countries deviate from the normal pattern and exhibit either positive or low correlation coefficients. These 12 countries coincide exactly with those that result to be anomalous based on the MDS maps. This analysis hence strengthens our conclusions, allowing us to reject the objection expressed in point 3 of the Reviewer: “Doesn't it all depend on the expansion of the output of these countries?” No, it does not, because anomalous countries show an anomalous relationship between output and self-citation rates.

To include these new results in the paper, the following Figure was added to the paper as Figure 5 with the following caption:

**Figure 5.** $\boldsymbol{S}\boldsymbol{R}^{\boldsymbol{I}}$ **and** $\boldsymbol{S}\boldsymbol{R}^{\boldsymbol{II}}$ **versus publication output by country.** Each data point represents data for a year between 1996 and 2019. Pearson correlation coefficients (by country and indicator) are overlaid.

The following lines were added:

[after line 431] Both indicators result to be highly correlated with the logarithm of the publication output for most of the countries (Fig. 5). Notably, the relationship between the self-citation rate (of both Types) and scientific production is of inverse proportionality: across most of the countries observed, an increase in scientific output is associated with a decrease in the self-citation rate. This contrasts with the direct proportionality found in prior studies [30, 40, 41, 46]. Interestingly, however, a few countries deviate from this general pattern, exhibiting either positive correlation (China for $SR^{I}$, Indonesia on both $SR^{I}$ and $SR^{II}$) or mild to low correlation coefficients (Colombia, Egypt, India, Iran, Italy, Malaysia, Pakistan, Romania, Russian Federation, Saudi Arabia, Thailand, Ukraine). The self-citation rate trend within this latter group of anomalous countries cannot be effectively predicted based solely on the trend of scientific production. (The scientific output trends of the 50 countries are reported in the Supporting Information.)

To better investigate differences between groups of countries, the correlation-based distance was used to produce […]

[after line 450] Note that the countries in the two latter zones are identical to those showing anomalous correlations with trends in scientific output.

[after line 509] Note that the anomalous self-citation rates of these countries cannot be explained by the trend of their scientific production, as evidenced by the low correlation coefficients found (Fig. 5). This suggests searching for specific perturbations of the citation behavior.

In the Supporting Information, the following figure S7 was added with the publication trends of the 50 countries:

To finally address the critical points of the Reviewer, we report here the trend of Italy against that of the other G10 countries, on the three indicators that include in their definition the publication share of a country. (Note that the publication share is a proportion, not an absolute count as in the scatterplots above. As a proportion of the world total, it is affected by the publication output of all countries in the world). Even if we believe that these indicators are less interpretable than self-citation rates, as explained in the paper (lines 295-309), they all confirm the anomaly of Italy. We highlight that all these indicators have been included in the Supporting Information to make them available to the research community.

4) “When bibliometric indicators are integrated into systems of incentives, they cease to be neutral measures to become active components in the research system. As such, they are able to modify the behavior of entire scientific communities. Hence, they should be handled by science policy makers with the most extreme caution”. Correct and agreeable statement. But then again, it MUST be so, for any metric implemented in an organised system to incentivise some behaviours (e.g., productivity increase), even with the awareness that this may induce collateral effects.

In our Conclusions, we advise science policymakers to handle bibliometric indicators with caution, we do not propose a ban of bibliometric indicators. Our advice is in line with guidelines designed to promote the responsible use of research metrics and indicators in the evaluation of scientific research, such as the Leiden Manifesto. Thus, we do not think that a modification of our Conclusions is required.

5) Table 2 caption: "Citing sources include all types of documents." What about cited ones? Wouldn't it be worth limiting the analysis to journal articles only?

The cited items include the same document types of the Publications column in Table 2 (articles, reviews, and conference papers), i.e., only the citations landing on those publications were counted. Focusing on journal articles only would penalize research fields where conference proceedings are key output, such as computer science. To be more precise, we modified the caption of Table 2 as follows:

The overline indicates the mean. Publications include only research articles, reviews, and conference papers. Citations (C) and self-citations (SI and SII ) are computed on a 2-year citation window. Citing sources include all types of documents, cited publications include research articles, reviews, and conference papers. Years: 1996-2019.

Future research may investigate whether focusing on journal articles only would significantly change the picture.

6) What about field dependency of citation distributions? It is not real to assume that all the top 50 analysed countries are equal in terms of field specialisations. As for the intertemporal analysis, if a country's mix of scientific (input) output changes (perhaps because of that country's policy), the overall citation pattern also changes. At least one brief comment on this limitation should be inserted in the paper.

We thank the Reviewer for the interesting suggestion on how to extend our research in the future. We agree that different scientific fields are likely to present different self-citation tendencies (as our 2019 study, cited as reference [26] in the paper, has shown). To mention these possible developments, the following lines were added in the Conclusions after line 766:

In future research, it will be interesting to further investigate the effects of policy pressure. For instance, by examining whether research fields characterized by varying epistemological, social, and institutional features respond differently to science policies. Or by investigating whether science policies may affect countries’ mix of scientific output, inducing specialization in areas rewarded by citation metrics (e.g., the medical sciences) at the expense of less “performing” areas (e.g., the humanities).
~~From this point of view,~~ On its part, this study contributes to the ongoing discussion on research evaluation systems by showing that when bibliometric indicators are integrated into systems of incentives, they cease to be neutral measures to become active components in the research system. As such, they are able to modify the behavior of entire scientific communities. Hence, they should be handled by science policy makers with the most extreme caution.
