# Supplementary material for: A global exploratory comparison of country self-citations 1996-2019
Source: PLoS One. 2023 Dec 29;18(12):e0294669. doi: 10.1371/journal.pone.0294669 (PMC10756561; doi:10.1371/journal.pone.0294669)
Supplement: Attachment — Submitted filename: Comments on R1.docx [file pone.0294669.s004.docx]

Here are my comments (in standard formatting) on the authors’ replies (in light blue).

The authors' replies to my comments are unsatisfactory and, in some cases, even a little disturbing.

“Self-reference” and “self-citation” are two different names for the *same* citation link in the citation network”: there was no need to explain it. Perhaps the authors did so because they either thought the reviewer was inexperienced or that the reviewer's contrarianism could be debarred through the instrumental use of obviousness.

“As discussed at length above, a self-citation is a self-reference seen from the viewpoint of the cited publication and, alternatively, a self-reference is a self-citation seen from the viewpoint of the citing publication. Hence, there is no contradiction in saying that researchers can use self-citations strategically”.

The contradiction is ontological, because self-citations are only observable, while self-references are controllable.

“Calling a citation link a self-reference or a self-citation is only a matter of perspective.”

“The distinction between a citation *given* and a citation *received* by an author (or any other bibliographic entity considered) is only a matter of viewpoint”.

I agree, I had only asked the authors to adopt the correct "viewpoint," i.e. a self-reference approach and not a self-citation approach, for the reasons I argued at length.

Unfortunately, the authors, on the one hand, admit the soundness of these arguments (“the Reviewer’s reasoning seems *prima facie* compelling”), on the other hand, however, they defend their choice, stating that “A closer look, however, shows that such a reasoning, albeit intuitive, is based on a misunderstanding”.

There is no misunderstanding at all: the identification of opportunistic behaviour comes through observation of the behaviour itself, not that of its potential "effects". If a person brings home an "anomalous" amount of apples, can he or she be suspected of stealing them?

Moreover, the disquisition about the alleged superiority of the authors' proposed approach over the one indicated by the reviewer is completely misplaced.

“Still, one may object that the interesting indicator are *not* self-citations/self-references *per se* but rather self-citations/self-references divided by some relevant quantity, i.e., self-citation *rates* (the indicator chosen in our study) and self-reference *rates* (the indicator that the Reviewer seems to have in mind) … “

I do not have this indicator in mind; it is a completely unfounded presumption, and, therefore, the whole subsequent defence by the authors drops.

The indicator I have in mind has no denominator but is a response variable "exposed" to a factor that can impact it: self-references in fact are a function of the publications an author has in his or her portfolio and that he or she can actually self-cite. I will return to this later regarding Figure 5.

“As explained at length in the methodological sections of the paper, there is no unique method for computing country self-citations for international publications, as there is no unique method for computing author self-citations for multi-authored publications”.

In principle, I’m okay with it, but the authors will agree that there are better methods than others, depending on what one wants to observe and analyse. Regarding the use of SR_I_, the error from which it is affected is obvious. I am not going to repeat myself here; it would be offensive. The authors' defence calling into question the fact that it is implemented in SCIVAL and, therefore, easy to replicate is unacceptable; it has little to do with the rigour of the scientific approach.

Finally, a short comment on the new elaborations introduced by the authors and, in particular, on Figure 5.

The fact that they find an "inverse proportionality" between output and self-citation for most countries is a sign that something is wrong. With a proper approach, i.e., based on self-references and a minimal lag between the two time series, the correlation must necessarily be positive. Assuming proper (and not opportunistic) use of self-references, it is clear that a given entity (a single author, for instance) will tend to increase her self-references proportionately to her output, i.e., the number of publications she can self-cite.
