# Supplementary material for: A global exploratory comparison of country self-citations 1996-2019
Source: PLoS One. 2023 Dec 29;18(12):e0294669. doi: 10.1371/journal.pone.0294669 (PMC10756561; doi:10.1371/journal.pone.0294669)
Supplement: Attachment — Submitted filename: Responses to the Reviewers_2.docx [file pone.0294669.s005.docx]

**Responses to the Reviewers**

*Please find our answers to the Reviewers’ remarks in the following. Reviewers’ comments are in standard formatting, our answers are in light blue. Changes to the paper’s text are indented, with added lines highlighted in yellow.*

# **Reviewer #1**

The response produced by the authors has taken account of the many criticisms levelled by the reviewers, both in terms of form and content.

The authors have produced new results to answer the questions raised by the reviewers. They have also produced a more detailed explanation of their approach, and less polemic around the issue of self-citation and self-referencing. Nevertheless, there are still divergent points of view on this study. The authors' response seems to me to be part of the scientific debate, It is for this reason that I agree to the publication of this paper.

It is important that the data from this study be made available for reproducibility and study purposes, in order to continue the scientific debate which is in the process of being established.

We would like to express our gratitude to the Reviewer for their supportive comments on our paper. We acknowledge that the topic is open to debate, and we hope the publication of our study will foster a broader discussion within the scientific community.

# **Reviewer #2**

The authors' replies are unsatisfactory and, for this reason, I'm suggesting rejection of the manuscript.

My comments are in the attached file.

*[We report here the attached file]*

Here are my comments (in standard formatting) on the authors’ replies (underlined).

The authors' replies to my comments are unsatisfactory and, in some cases, even a little disturbing.

“Self-reference” and “self-citation” are two different names for the same citation link in the citation network”: there was no need to explain it. Perhaps the authors did so because they either thought the reviewer was inexperienced or that the reviewer's contrarianism could be debarred through the instrumental use of obviousness.

We have the utmost respect for the knowledge, experience, and skills of the Reviewer. With our reply, we aimed only to be as clear as possible in order to avoid any possible misunderstanding. We apologize if our approach came across as disrespectful.

“As discussed at length above, a self-citation is a self-reference seen from the viewpoint of the cited publication and, alternatively, a self-reference is a self-citation seen from the viewpoint of the citing publication. Hence, there is no contradiction in saying that researchers can use self-citations strategically”.

The contradiction is ontological, because self-citations are only observable, while self-references are controllable.

Unfortunately, we are unable to fully understand what the Reviewer means by “ontological”. As we explained at length, a self-citation and a self-reference refer both to the same link in the citation network. They are “ontologically” equivalent since they refer to the same entity, exactly as – to quote the famous example from Frege – the Morning Star and the Evening Star are “ontologically” the same as they are just different names for the same celestial body, Venus. As such, they can *both* be controlled (by authors) and observed (by analysts).

“Calling a citation link a self-reference or a self-citation is only a matter of perspective.”

“The distinction between a citation given and a citation received by an author (or any other bibliographic entity considered) is only a matter of viewpoint”.

I agree, I had only asked the authors to adopt the correct "viewpoint," i.e. a self-reference approach and not a self-citation approach, for the reasons I argued at length.

Unfortunately, the authors, on the one hand, admit the soundness of these arguments (“the Reviewer’s reasoning seems prima facie compelling”), on the other hand, however, they defend their choice, stating that “A closer look, however, shows that such a reasoning, albeit intuitive, is based on a misunderstanding”.

There is no misunderstanding at all: the identification of opportunistic behaviour comes through observation of the behaviour itself, not that of its potential "effects". If a person brings home an "anomalous" amount of apples, can he or she be suspected of stealing them?

Once more, we find ourselves struggling to fully comprehend the Reviewer’s point. Indeed, if someone brings home an anomalous amount of apples, there are reasons to inquiry how they obtained those apples. Stealing is one of the possible explanations, clearly not the only one. More generally, the observations made by the Reviewer appear to contradict elementary investigative practices, particularly in legal proceedings. When determining whether a specific behavior occurred in the past (let’s say a murder), a detective indeed gathers clues (e.g., blood stains), which count as evidence precisely because they can be plausibly linked causally to the event in question. If the behavior were observable directly, there would be no need for an investigation in the first place. This type of reasoning, which is the same applied in our study, is called in logic “inference to the best explanation” (see <https://plato.stanford.edu/entries/abduction/> )

Moreover, the disquisition about the alleged superiority of the authors' proposed approach over the one indicated by the reviewer is completely misplaced.

“Still, one may object that the interesting indicator are not self-citations/self-references per se but rather self-citations/self-references divided by some relevant quantity, i.e., self-citation rates (the indicator chosen in our study) and self-reference rates (the indicator that the Reviewer seems to have in mind) … “

I do not have this indicator in mind; it is a completely unfounded presumption, and, therefore, the whole subsequent defence by the authors drops.

The indicator I have in mind has no denominator but is a response variable "exposed" to a factor that can impact it: self-references in fact are a function of the publications an author has in his or her portfolio and that he or she can actually self-cite. I will return to this later regarding Figure 5.

We thank the Reviewer for clarifying to us that the indicator they have in mind is the raw number of self-references, without any kind of normalization. They clarify also that they propose a model where the raw-number of self-references depends on publications (we hope to interpret correctly, since, formally, the number of publications is not properly a factor but a discrete variable). The aim of our paper is mainly exploratory, and we do not propose a model approach to the problem of country self-citation. Our main aim is to contribute to the stream of literature reviewed in Section 2 where the main focus is to build suitable indicators for comparing self-citations among different entities. In this context, none has ever proposed the raw number of self-references, since it is clearly a not normalized indicator.

Obviously enough, $SR^{I}$ or $SR^{II}$ can be both introduced as “response variables” in models where the number of publications is an explanatory variable, or better one of many exploratory variables. For example, Seeber and coauthors (<https://doi.org/10.1016/j.respol.2017.12.004>) used the self-citation rate as the response variable in a model developed for checking the robustness of another model where the response variable is the number of self-citations per paper.

“As explained at length in the methodological sections of the paper, there is no unique method for computing country self-citations for international publications, as there is no unique method for computing author self-citations for multi-authored publications”.

In principle, I’m okay with it, but the authors will agree that there are better methods than others, depending on what one wants to observe and analyse. Regarding the use of SR_I_, the error from which it is affected is obvious. I am not going to repeat myself here; it would be offensive. The authors' defence calling into question the fact that it is implemented in SCIVAL and, therefore, easy to replicate is unacceptable; it has little to do with the rigour of the scientific approach.

We respectfully hold a different view from the Reviewer on this matter. The availability of an indicator on a platform widely used around the world facilitates scrutiny of our results by the scientific community. We have discussed the strengths and weaknesses of $SR^{I}$ at length in the paper, allowing readers to form their own opinions. Moreover, in another paper from us, we delve even deeper into the analysis of the same indicator.

Finally, a short comment on the new elaborations introduced by the authors and, in particular, on Figure 5.

The fact that they find an "inverse proportionality" between output and self-citation for most countries is a sign that something is wrong. With a proper approach, i.e., based on self-references and a minimal lag between the two time series, the correlation must necessarily be positive. Assuming proper (and not opportunistic) use of self-references, it is clear that a given entity (a single author, for instance) will tend to increase her self-references proportionately to her output, i.e., the number of publications she can self-cite.

We kindly invite the Reviewer to adopt what they deem to be a “proper approach” and verify their prediction, which, as of now, stands as an hypothesis requiring validation against data. We are confident that such a study would constitute a valuable contribution to the debate on self-citations.

# **Reviewer #3**

The present manuscript is an investigation into temporal developments into country self-citations, especially in relation to science policies at national level which involve citation metrics. Self-citations are simple but also easily observed means to strategically influence citation metrics. This study provides an extensive review of country-level self-citation methodology and continues to report on country-level values. The study is overall well designed and conducted. I have only a few remarks to the authors to possibly improve their paper.

We thank the Reviewer for the positive assessment of our study.

In their choice of two country self-citation indicators, the rationale for the Type II indicator is completely clear but that for the Type I indicator is not. This indicator's definition and logic seem to contradict the very meaning of the term "country self-citations" even though it involves self-citations and countries. In this indicator, as I understand it, all co-authors' citations of a target paper are considered. For all countries involved in the target paper, any co-author self-citation is counted, regardless of that author's country. This means both same-country author self-citations and different-country self-citation are counted, contrary to the Type II indicator. I do not follow how a different country's author self-citations can possibly be called country self-citations.

We acknowledge that the definition of country self-citations of Type I can produce counterintuitive results when applied to multi-authored papers that are produced by authors from different countries. Let us say that paper $p_{1}$ is co-authored by author $a_{1}$ from the United States and author $a_{2}$ from France. If $p_{1}$ is cited by $p_{2}$, which is authored by $a_{1}$ from the United States and $a_{3}$ from Germany, then the citation $p_{2}\to p_{1}$ will count as a country self-citation of Type I for the United States and also for France. This is the inevitable consequence of defining country self-citations as any citations between papers whose sets of countries *overlap*. (Note that this is the definition of country self-citation used in SCIVAL, see the reference [44] in the paper). In the citation network reported in Figure 2 of the paper, a scenario of this kind happens with publication number 1, which is the result of an international collaboration between Italy and the Netherlands. As shown in Table 1, the citation link 1-6 counts as a country self-citation of Type I *both* for Italy and the Netherlands. It seems indeed odd to count this citation among the country self-citations of the Netherlands, as the citing paper number 6 has in fact no Dutch author. Still, since the set of countries of paper number 1 *overlaps* with the set of countries of paper number 6, then the citation link 1-6 must be counted as a country self-citation also for the Netherlands.

The necessary but apparently counterintuitive nature of this conclusion arises from the fact that international papers must be attributed to *all* the collaborating countries. Hence, a citation can count as coming from a *different* country *only when* it comes from a paper whose countries do not overlap at all with those of the cited paper, as citation 4-1 in Figure 2. In fact, the longer the list of countries involved in a paper, the higher the chances that citations landing on that paper will be country self-citations of Type I in this counterintuitive sense. In the extreme case of the fictious country “World” that includes all the countries on the planet, any citation to the paper of the “World” will count as a country self-citation of Type I, i.e., the “World” has $SR^{I}=1$, as discussed in the paper number [26] cited in the bibliography.

We note, however, that the Reviewer’s comment on the definition of country self-citation of Type I (“For all countries involved in the target paper, any co-author self-citation is counted, regardless of that author's country”) is slightly incorrect. Author’s countries are precisely what determines if a citation is a country self-citation depending on whether they overlap or not. In fact, if an author *changes* country of affiliation between her cited and citing publications, her self-citations would count as an author self-citations but *not* as country self-citations. For instance, let’s say that author $a$ is affiliated with country $c_{1}$ at time $t_{0}$ and produces publication $p_{1}$ at $t_{0}$. At time $t_{1}$, $a$ changes affiliation to country $c_{2}$ and produces publication $p_{2}$ that cites $p_{1}$. In this scenario, the citation $p_{2}\to p_{1}$ would count as an author self-citation of $a$ but not as a country self-citation of Type I for $c_{1}$ or $c_{2}$.

To further clarify this point, the paragraph beginning on line 272 was modified as follows:

This happens because country self-citations Type I of international publications include the citations coming from *any* of the collaborating country. In fact, a paper resulting from the collaboration of numerous countries is more likely to attract self-citations of Type I in comparison to a paper resulting from the collaboration of only a few countries. This is because there is a higher likelihood that the countries of the citing papers will overlap with the countries of the former rather than with the countries of the latter (see [26], sec. 3). In the extreme hypothetical scenario of a paper resulting from the collaboration of all countries in the world, *any* citation to this paper would be considered a self-citation of Type I, as the country of the citing papers will always overlap with at least one of the countries of the cited paper. ~~This means, however, that international publications tend to inflate the country self-citations (see [26], sec. 3).~~ Country self-citations of Type II are introduced precisely to correct this somehow counterintuitive property of country self-citations Type I. Country self-citations of Type II in fact include only the citations coming from the focal country.

Notwithstanding these counterintuitive properties of country self-citations of Type I, they remain a valuable research objects in our perspective because, differently from country self-citations of Type II, they are sensitive to a country’s internal citation exchange. The discovery that countries like China exhibit divergent trends between country self-citations of Type I and II represents a significant outcome of our study, in our view. Naturally, we remain receptive to the possibility that future research may provide alternative explanations for these observed trends.

Regarding the discussion of results: if the countries identified as anomalous in terms of self-citation indicators "all share a common trait, namely the introduction of direct or indirect rewards for the bibliometric performance of scientists" (p. 27), is the opposite also true? Namely, is it the case that all the other countries do not have national policies involving bibliometrics? This is an important further consideration. If there are such countries with citation-based incentives and they don't show anomalous country self-citation developments, this would seriously change the interpretation of results.

We thank the Reviewer for raising this key point about the conclusions of the study. We acknowledge that our results show that all anomalous countries are characterized by performance-based funding systems that use bibliometric incentives but *not* that *only* the anomalous countries share this trait. In fact, there are some countries that use bibliometrics to evaluate the performance of their universities but do not result anomalous under the profile of self-citations. We have therefore recalibrated our conclusions, discussing a further condition that would render a country anomalous, namely the proximity of the citations-based incentives to the individual careers or wage of researchers.

The following paragraph was therefore added after line 795 in the Conclusions:

Remarkably, our results show that merely having a performance-based funding system involving citation-based indicators is insufficient for a country to exhibit anomalous self-citation behavior. For example, countries like Belgium, Bulgaria, Croatia, and Sweden employ citation-based indicators in evaluating their university systems, yet they do not display anomalous self-citation tendencies [Jonkers & Zacharewicz 2017]. It appears that the crucial factor influencing this anomalous self-citation behavior is the *proximity* of incentives based on citations to an individual researcher's career and wage. It seems that the closer these incentives are to affecting researchers' career progression and wages, the more likely they are to influence citation behavior. In Italy, for instance, the national scientific qualification, which is awarded based on citation indicators, has a direct impact on the career of researchers, as without it researchers cannot access tenure-track positions or advance to full professorships. By contrast, in countries where citation-based indicators contribute to complex funding formulas at the university level, the influence on citation behavior is more distant from individual researchers and, consequently, less significant.

In future research, it will be important to further investigate how varying architectures of performance-based funding systems have more or less impact on citation behavior, exploring in particular the extent to which the proximity of citation-based incentives to individual careers and wage induces strategic behavior. ~~it will be interesting to further investigate the effects of policy pressure. For instance, by~~ Other promising topics for future research include examining whether research fields characterized by varying epistemological, social, and institutional features respond differently to ~~science~~ citation policies~~. Or by~~ and investigating whether ~~science policies~~ citation incentives may affect countries’ mix of scientific output, inducing specialization in areas rewarded by citation metrics (e.g., the medical sciences) at the expense of less “performing” areas (e.g., the humanities). On its part, this study contributes to the ongoing discussion on research evaluation systems by showing that when bibliometric indicators, and citation-based indicators in particular, are integrated into systems of incentives, they cease to be neutral measures to become active components in the research system. As such, they are able to modify the behavior of entire scientific communities. Hence, they should be handled by science policy makers with the most extreme caution.

And the following report of the European Commission was added to the reference list:

Jonkers, K. & Zacharewicz, T., Research Performance Based Funding Systems: a Comparative Assessment, Publications Office of the European Union, Luxembourg, EUR 27837 EN, ISBN 978-92-79-68715-0, doi:10.2791/70120, JRC101043.

Finally, the reference list of this paper is poorly quality checked and should be very carefully revised. Only looking superficially I found the following faulty references and there may well be more:

69. Abalkina A. type [; 2021]Available from: https://scholarlykitchen.sspnet.org/2021/02/04/guest-post-unethical-practices-in-research-and-publishing-evidence-from-

83. Gill J. type [; 2008]Available from: https://www.timeshighereducation.com/news/malaysian-rankings-flop-shames-the-nation/404570.article.

85. Malaysia MoEo. type [; 2007]Available from: https://www.ilo.org/dyn/youthpol/en/equest.fileutils.dochandle?p_uploaded_file_id=477.

113. ur Rahman A. Building a Knowledge Economy. In: Baydoun E, Hillman JR,editors. Universities in Arab Countries: An Urgent Need for Change:Underpinning the Transition to a Peaceful and Prosperous Future. Cham:Springer International Publishing; 2018. p. 105–121. Available from:https://doi.org/10.1007/978-3-319-73111-7_5.

134. for Science Innovation D, Technology U. type [; 2022].

We are grateful to the Reviewer for carefully checking the list of references. We corrected the mistakes throughout the entire bibliography of the paper.

These issues notwithstanding, I think the paper already meets the criteria for being published. I would thus recommend that the authors seriously consider them.
